# Processing speed predicts SuperAging years later

**DOI:** 10.1186/s40359-023-01069-7

**Published:** 2023-02-02

**Authors:** Zuzana Ticha, Hana Georgi, Ben Schmand, Radek Heissler, Miloslav Kopecek

**Affiliations:** 1 Prague College of Psychosocial Studies, Hekrova 805, 149 00 Prague 11, Háje, Czech Republic; 2 Department of Psychology, University of Amsterdam, Amsterdam, The Netherlands; 3 National Institute of Mental Health, Klecany, Czech Republic; 4 Department of Psychiatry and Medical Psychology, Third Faculty of Medicine, Charles University, Prague, Czech Republic

**Keywords:** Aging, Healthy, Cognition, Superagers

## Abstract

**Background:**

SuperAging is one of the current concepts related to elite, resilient or high-functioning cognitive aging. The main aim of our study was to find possible predictors of SuperAgers (SA).

**Methods:**

Community-dwelling older persons (*N* = 96) aged 80–101 years in 2018 were repeatedly tested (year 2012 and 2018). SA were defined based on their performance in 2018 as persons of 80+ years of age who recalled ≥ 9 words in the delayed recall of the Philadelphia Verbal Learning Test, and had a normal performance in non-memory tasks [the Boston Naming Test, the Trail Making Test Part B, and Category Fluency (“Animals”)], which was defined as a score within or above one standard deviation from the age and education appropriate average. Three composite scores (CS; immediate memory, processing speed, and executive functions) were created from the performance in 2012, and analysed as possible predictors of SA status in 2018.

**Results:**

We identified 19 SA (15 females) and 77 nonSA (42 females), groups did not significantly differ in age, years of education, and sex. The logistic regression model (*p* = 0.028) revealed three predictors of SA from the baseline (year 2012), including processing speed (*p* = 0.006; CS-speed: the Prague Stroop Test—Dots and the Digit Symbol Substitution Test), sex (*p* = 0.015), and age (*p* = 0.045).

**Conclusions:**

Thus, SA may be predicted based on the level of processing speed, which supports the hypothesis of the processing speed theory of healthy aging.

## Background

Longer life expectancy and global aging of societies promote research of the spectrum of cognitive aging, which directly affects self-sufficiency and indirectly economic and labour issues related to both formal and informal care [1–3].

After decades of research of pathological cognitive decline, an area of research of cognitive resilience, elite cognitive aging or SuperAging has emerged. Definitions of superior cognition in old age vary, but each has precise criteria [4]. Most studies of superior or resilient cognitive aging use individuals of 65 years or younger as reference groups, which from a biological point of view is a better design than using an age-appropriate reference group, since the decline of cognitive functions and the thinning of brain regions through aging is not linear but rather non-linear [4–8].

The term SuperAging was coined by the Northwestern Mesulam Center for Cognitive Neurology and Alzheimer’s Disease. SuperAgers (SA) were defined as individuals over the age of 80 with episodic memory performance at least as good as normative values for 50–65-year-olds, while their non-memory performance is on the mean level of their peers [9, 10]. In other words, we might say that SA are younger in memory age by at least 15 years.

The prevalence of SA in general population is, to our knowledge, unknown. However, several studies, including the one by our team, found about 12–35% of healthy aging old- and oldest-old adults to be SA [11–13]. Previous research of SA focused especially on the investigation of anatomic, genetic, and histopathologic markers. SA have youthful brain regions in the major paralimbic and limbic nodes of the default mode and salience networks that support attentional, executive, and mnemonic processes subserving memory function [9, 10, 14–19]. Also, preserved greater neural differentiation and reinstatement helps SA with superior memory performance [17]. However, non-memory cognitive performance and other associated phenomena of SA has not been of much focus [10, 15, 16, 19]. Despite it, there is a study [16] suggesting, that SA in comparison to their peers have better performance in other cognitive domains a few years earlier, such as working memory or processing speed. They pointed out that despite the better performance in these domains, it declines in the same rate as in normal older adults (i.e. the cognitive maintenance does not differ). Thus, it seems that SA are somehow protected from the decline of episodic memory, but not from the decline in other cognitive domains.

Processing speed is one of the cognitive functions that declines with the age and it describes how fast a person executes mental operations in order to complete a task [20]. It is considered as one of the strongest predictors of performance in different cognitive tasks in older adults [20–22]. Its decline begins from midlife and linearly continues with increasing age [23–26]. Significant slowing in processing speed is associated with the overall cognitive decline and subsequently with the need for help with the activities of daily living [22, 27, 28], clinical disorders of cognition, mobility and mood [29], or even with mortality [30, 31]. On the other hand, there are factors related to a robust and stable processing speed in older age, such as greater self-reported physical activity [23], that has also been reported in SA [32]. Or despite the same amount of physical activities, it seems that SA perceive it as less demanding compared to nonSA [33].

As described above, SA are identified based on their superior memory performance and age appropriate non-memory cognitive performance (naming ability and executive functions) at a certain point beyond the age of 80. Previous studies on aging showed that faster processing speed is associated with better performance in other cognitive domains (i.e. memory). These studies used data from one time point using different samples (cross-sectional [19]) or longitudinal data for the evaluation of trajectory [16, 34, 35]. In contrast, we decided to use longitudinal approach not to analyse the trajectory but in order to seek the best predicting model of SA. Data from a time point (year 2012) were used to predict SA status determined at a later time point (year 2018). Therefore, the former time point (year 2012) rendered the baseline performance and predictors, and the performance at the latter time point (year 2018) was the dependent variable for determining of SA. We hypothesized that processing speed is the best predictor of SA status in comparison to other cognitive domains such as executive functions and short-term memory. To our knowledge this is the first study that aims to find the best predictor of future status of SA.

## Method

### Study design and sample

The current study uses the data from the project Cognitive SuperAging (CoSA) realized in 2018–2020, and from the first wave of the National Normative Study of Cognitive Determinants of Healthy Aging (NANOK), which was its predecessor, realized in 2012–2015 [36], and approved by the Ethics Committee of the Prague Psychiatric Centre under the reference number 64/11. Only participants of the NANOK were invited to the CoSA. The inclusion criteria for the NANOK were an age of ≥ 60 years, Czech as first language, willingness to participate in the 4-years study, and a medical history free of cognitively critical issues (such as a diagnosis/treatment for a serious neurological disorder, stroke, traumatic brain injury, acute phase of a serious mental disorder, hospitalization for a substance abuse, or chemotherapeutic treatment). There were 568 persons enrolled in wave 1 (the year 2012), aged 60–98 years, who met the inclusion criteria. The assessment and recruitment (through advertising in the local media, doctor's waiting rooms, post offices, senior institutions, or on the web), took place in 12 regions of the Czech Republic to increase representativeness, with the help of 25 trained psychometrists. Then, in the CoSA (the year 2018), there were 113 persons enrolled from the NANOK who met the inclusion criteria (age ≥ 80) and provided their informed consent. For this study, five participants were excluded due to Mini-Mental State Examination (MMSE) < 23 suggestive of an imminent cognitive disorder; four persons were excluded due to the age limit—the persons declared to be 80 during recruitment when actually they were not yet 80 at the assessment date; one participant was excluded because of their inability to finish the protocol; seven participants were excluded because they did not finish Trail Making Test (part B), a test included in the definition of SA. Therefore, the final dataset for analysis included 96 participants aged from 80 to 101 years (mean age 86.32, *SD* = 5.06), 40.6% were male, and the sample had an average of 13.58 years (SD = 3.92) of education. Demographic data are in Table 1.

**Table 1:** Demographic data and performance in the follow-up (2018)—comparison of SA versus nonSA

Follow-up characteristic	SA (*n* = 19)		NonSA (*n* = 77)		Cohen’s *d*	*p*
	*M* (*SD*)	Min–Max	*M* (*SD*)	Min–Max		
Age in years	84.68 (5.06)	80–98	86.73 (5.01)	80–101	0.41	0.115
Education in years	13.11 (3.07)	9–19	13.70 (4.11)	6–28	0.15	0.560
MMSE	28.16 (1.54)	25–30	26.75 (1.99)	23–30	0.74	**0.005**
PVLT-DR	10.32 (1.16)	9–12	6.42 (2.80)	0–11	1.52	**< 0.001**
BNT-30	27.90 (1.66)	25–30	24.90 (4.67)	8–30	0.70	**< 0.001**
TMT-B	125.10 (59.06)	45–241	177.27 (126.26)	64–912	0.45	0.083
CF-animals	23.10 (6.53)	12–39	17.79 (5.72)	8–36	0.90	**0.001**

*SA* SuperAgers, *nonSA* non-SuperAgers, *MMSE* Mini-mental state examination, *PVLT-DR* Philadelphia verbal learning test—delayed recall, *BNT-30* Boston Naming Test-30, *TMT-B* Trail making test—part B, *CF-Animals* Category verbal fluency (Animals)

*p*-values of all comparisons (SA vs. nonSA) reaching statistical significance (*p* < 0.05) are written in bold

### Protocol approval and consents

The study was approved by the Ethics Committee of the National Institute of Mental Health, Klecany, Czech Republic (reference number 115/17) and all participants signed an informed consent form.

### Transparency and openness

We describe all manipulations and measures that were collected. Data that met our a priori exclusion criteria (described above) were excluded. Analyses were performed using IBM SPSS 23 and JASP 0.14.1. Deidentified data are available at the project’s Open Science Framework page at osf.io/wbcer/. This study’s design and its analysis were not pre-registered.

### Procedure and instruments

The assessment protocol consisted of a battery of neuropsychological tests [30]. For this study, we analysed measures used in both the CoSA and NANOK. Performance in the baseline (cognitive measures without those used in SA definition) was used as the predictor of the SA status, and measures in the follow-up were used for the identification of SA, and description of the present state.

### Baseline performance (2012): predictors of SA

- *Short-term memory (Composite score-memory (CS-memory))*: the Wechsler Memory Scale-Revised subtest of Logical Memory IA (immediate recall) [37, 38], and the Digit Span Forward (number of correctly recalled sequences) [39].
- *Processing speed (CS-speed)*: the Digit Symbol Substitution Test (number of correctly drawn symbols in 120 s [39]) and the Prague Stroop Test (PST) subtest Dots (completion time in s [40]).
- *Executive functions (CS-executive)*: the Prague Stroop Test subtest Colors (completion time in s [40]), and the Digit Span Backward (number of correctly recalled reversed sequences [39]).

### Follow-up measures (2018): general and SA status

- *General screening*: the Mini-Mental State Examination (MMSE; [41, 42]).
- *Identification of SA*: We identified SA in year 2018 according to the following procedure. The mean delayed recall score of the Philadelphia Verbal Learning Test (PVLT-DR) was 9 words for the age group 60–64 years [43]. The score of 9 words is equal to the score used in the previous SuperAging studies, where it represented the average normative value of the delayed verbal recall score of the Rey Auditory Verbal Learning Test (another word list test) for individuals in their 60 s [10, 14, 16, 19]. Furthermore, additional criteria for SA in accord with the previous SuperAging studies were applied: that is, performance on non-memory tasks such as the Boston Naming Test (30-item) (BNT-30; [44, 45]), the Trail Making Test—part B (TMT-B; [46, 47]), and Category (Animals) Fluency (CF-Animals; [48]) to be equal or better than minus one standard deviation for their age band [9, 10, 14, 15, 19, 45]. The performance in the SA criterion tasks is in Table 1.

### Statistical analysis

We created 3 composite scores (CS) of the immediate memory, processing speed, and executive functions by standardizing performance on each task, and averaging the z-scores. Z-scores for specific test were calculated using the mean and standard deviation (taking into account age, sex, and education) computed from normative data of older adults [36]. We chose to use this larger group as the z-score reference cohort instead of the study cohort for increased accuracy of the mean and variance estimates [16]. Additionally, interference score of the PST was calculated using ratio of two subtests: Colors/Dots. Two ratio interference scores were calculated, first using raw scores, and second using standardized scores.

The group comparison of cognitive performance and demographic variables in 2012 was performed using independent sample t-tests. In addition to effect sizes, we also report Bayes factors (BF_10_) calculated in accordance with recommendations by Wetzels and Wagenmakers [49]. We report probability of our data fitting under the null vs. the alternative hypothesis. Note that values larger than one are in favour of the alternative hypothesis and the values smaller than one are in favour of the null [49].

Series of binomial logistic regression analyses (method: Enter) were performed to find predictors of the SA status in 2018 based on the baseline neuropsychological data (year 2012). Age, number of years of education, sex, and composite scores (each CS separately) were used as predictors in the regression model (method Enter); SA status in 2018 was the dependent variable.

Then, all significant CS predictors (*p* < 0.05) plus age, number of years of education, and sex were inserted in the binomial logistic regression (method: Stepwise) to identify the best model for predicting SA.

Moreover, analysis of associations between significant predictor (2012) and PVLT delayed recall in 2018 (Pearson’s correlation) and status of SA in 2018 (Rank Biserial correlation) were performed.

The level of significance was for all analyses set at 0.05.

## Results

### Factors associated with SuperAging (2018)

We identified 19 SA (18%) out of 96 persons. SA did not significantly differ in the number of years of education nor in the age (all *p* > 0.05; Table 1) from nonSA. We did not find a significant association between sex and status of SA (*p* > 0.05), 15 females and 4 males were in the SA group. Groups significantly differed in the MMSE (*Χ*^2^ = 3.76; *p* = 0.005), the mean score of SA was ca. 2 points higher than the mean of nonSA (Table 1).

### Baseline performance (2012)

SA compared to nonSA, as identified in the follow-up (year 2018), had significantly better performance at the baseline (year 2012) only in the CS-speed (*p* = 0.012) with medium effect size and substantial evidence in favour of the effect (BF_10_ = 4.06) (Table 2). Groups did not significantly differ in CS-memory nor in CS-executive (*p* > 0.05; Table 2) with substantial evidence in favour of null hypothesis (i.e. no difference between the groups; BF_10_ = 0.26). Despite non-significant difference between the groups in CS-executive, there was a medium effect size and anecdotal evidence in favour of the effect (BF_10_ = 1.17).

**Table 2:** Comparison of baseline (Year 2012) neuropsychological performance of SA versus nonSA

Measure (2012)	SA (*n* = 19)		nonSA (*n* = 77)		*t*	*p*	Cohen’s *d*	BF_10_
	*M*	*SD*	*M*	*SD*				
CS-memory	0.15	0.80	0.14	0.89	0.045	0.964	0.01	0.26
CS-speed	0.55	0.67	0.06	0.75	2.570	**0.012**	0.66	4.06
CS-executive	0.48	1.21	0.04	0.84	1.525	0.141	0.49	1.17

*N*, 96; SA, SuperAgers in year 2018; nonSA, non-SuperAgers in year 2018; CS, composite score; *t*, results of t tests; Cohen’s *d*, effect size; BF_10_, Bayes factor indicating the evidence in favour of an effect

All comparisons (SA vs. nonSA) reaching statistical significance (*p* < .05) are written in bold

Moreover, groups did not significantly differ in PST interference scores (raw score: *t*(94) = 0.898, *p* = 0.371, *d* = 0.23, BF_10_ = 0.37; standardized score: *t*(94) = − 0.393, *p* = 0.696, *d* = − 0.10, BF_10_ = 0.28) and there was anecdotal respectively substantial evidence in favour of the null hypothesis (i.e. no difference).

### Predictors of SuperAging

Logistic regression analysis was performed to identify significant predictors of SA. Firstly, we performed a series of separate logistic regression analyses (method: Enter), that were controlled for sex, age, and years of education. Based on these analyses, two measures were identified as significant predictors: CS-speed (*p* = 0.006) and CS-executive (*p* = 0.028). CS-memory was not identified as significant predictor (*p* > 0.05). Age was significant predictor when combined with all three CSs (CS-speed, CS-memory, and CS-executive), and in case of sex only in combination with CS-speed. There was no significant effect of the number of years of education. All results are presented in Table 3.

**Table 3:** Binomial logistic regression analysis (method: enter) predicting SA status

Measure (2012)	BIC	AIC	R^2^_MCF_	Measure 2012 *OR* [95% CI]	Sex *OR* [95% CI]	EDU-Y *OR* [95% CI]	Age *OR* [95% CI]
CS-memory	111	98	.08	1.03 [0.57, 1.87]	3.64 [0.97, 13.63]	0.99 [0.85, 1.16]	0.89 [0.79, 1.01]*
CS-speed	101	88	.18	3.81 [1.48, 9.84]**	4.94 [1.17 20.93]*	0.97 [0.82, 1.15]	0.86 [0.75, 1.00]*
CS-executive	105	93	.14	1.90 [1.07, 3.35]*	3.68 [0.92, 14.69]	0.98 [0.83, 1.15]	0.86 [0.75, .99]*

*N*, 96. BIC, Bayesian information criterion; AIC, Akaike information criterion; R^2^_MCF_, McFadden’s R squared; OR, Odds ratio; 95% CI, 95% confidence intervals; EDU-Y, years of education; CS, composite score

*p* values (*p < 0.05; **p < 0.01) indicate the statistical significance of a predictor independent of all other factors in the model

Then, we performed logistic regression analysis (method: Stepwise) to identify the best model for predicting SA. All significant measures from the previous analysis (CS-speed, CS-executive; see Table 3), number of years of education, sex, and age were entered in the stepwise logistic regression analysis. Compared to other significant models, model no. 3 (Table 4), which included the following measures: CS-speed (*p* = 0.006), sex (*p* = 0.015), and age (*p* = 0.045) was the best prediction model of SA (BIC = 96, AIC = 86, *p* = 0.028). Table 4 shows only the models that reached statistical significance. CS-executive did not reach it, therefore it is not included.

**Table 4:** Binomial Logistic Regression Analysis (Method: Stepwise) Predicting SA Status

Model	BIC	AIC	R^2^_MCF_	Measure (2012)	*p*	*OR* [95% CI]
1	98	93	.07		0.010	
				CS-speed	0.015	2.56 [1.19, 5.46]
2	97	89	.14		0.014	
				CS-speed	0.007	3.17 [1.37, 7.36]
				Sex	0.024	4.41 [1.21, 16.00]
3	96	86	.18		0.028	
				CS-speed	0.006	3.74 [1.47, 9.52]
				Sex	0.015	5.39 [1.40, 20.82]
				Age	0.045	0.87 [0.75, 1.00]

*BIC* Bayesian information criterion, *AIC* Akaike information criterion, *R*^*2*^_*MCF*_ McFadden’s R squared, *OR* Odds Ratio, *95% CI* 95% confidence interval, *CS* composite score

The results showed that CS-speed (*OR* = 3.74, 95% CI 1.47, 9.52) was an important predictor of the future status of SA, along with sex (*OR* = 5.39, 95% CI 1.40, 20.82), and age (*OR* = 0.87, 95% CI 0.75, 1.00). Moreover, CS-speed was not significantly associated with PVLT delayed recall in year 2012 (*r* = − 0.04, *p* = 0.728) nor in year 2018 (*r* = 0.10, *p* = 0.338). However, significant association between CS-speed and SA status (*r*_*b*_ = 0.26, *p* = 0.012) was revealed, which is in line with results of Binomial logistic regression analysis.

In closer look, each second faster in PST-D (while maintaining other variables on the same level) meant a 0.6 times (*OR* = 0.60, 95% CI 0.41, 0.89) higher chance of being SA. As for DSST-120, the chance of SA status increased by 1.13 times (*OR* = 1.13, 95% CI 1.03, 1.24) with every point. Also, women had higher chance of being SA compared to men. As well, with younger age increased the chance of being SA.

## Discussion

The current study aimed to identify the role of processing speed in the predicting of the status of SuperAger (SA). For this purpose, the longitudinal approach was chosen. Predictors were identified from the baseline data (2012) and data from the follow-up (2018) were used as the dependent variable to determine the status of SA. We found that processing speed measures (CS-speed) in combination with sex and age, predicted SA status.

The definition postulates that SA are superior in episodic memory compared to their peers [9, 10, 16], namely in delayed recall of a word list test such as Rey Auditory Verbal Learning Test (RAVLT) or PVLT. Despite it, SA did not significantly differ in other short-term memory measure (CS-memory) from nonSA. These results are not in accord with previous studies suggesting that SA had a better baseline episodic memory performance (measured with composite score) compared to their peers [16, 34]. The reason of this discrepancy could be that our CS-memory was composed from Logical memory test—Immediate Recall and Digit span (forward), which both test only immediate recall of the material without refreshing opportunities, or short-term memory, and do not support developing of a learning strategy unlike the word list tests or Logical Memory Delayed Recall that were used by Dang et al. [34] or Harrison et al. [16].

Our study revealed that SA significantly differed at baseline from nonSA in the non-memory measure: CS-speed, and the difference in CS-executive was with medium effect size but it did not reach significance. After controlling for age, sex, and education, CS-speed and CS-executive turned out to be predictors of future SA status (see Table 3). This is in accordance with previous studies [16, 35], but not with Dang et al. [34], who reported a better baseline performance of SA in executive functions but not in processing speed measures. Based on previous research, these two cognitive abilities (processing speed and executive functions) to some extent overlap, and tests measuring processing speed usually involve executive processes (e.g. DSST involves inhibition, shifting, and updating) [50]. In our case, we found that processing speed (CS-speed) is a best predictor of the future status of SA (see Table 4). Although, CS-executive seemed to also predict the future status of SA, but in the further regression analysis was not predictor strong enough in comparison with CS-speed. Also, both CSs (CS-speed and CS-executive) are significantly strongly associated (*r* = 0.60; *p* < 0.001), so possibly these measures may share mutual variance [50, 51]. Additionally, PST ratio interference scores were calculated (both using raw scores or standardized scores). There was non-significant difference between SA and nonSA in these measures. So, based on our results, it seems that processing speed and less executively demanding measures (DSST and PST-D) are stronger in predicting future SA status, as suggested also by Baudouin et al. [51].

Faster processing of information may have facilitated overall performance of SA, as it is suggested in our study. Processing speed is important for our everyday activities because it supports performance in other cognitive domains [20, 21]. Its slowing is associated with a decline in cognition, mobility, and mood [29, 52]. On the other hand, in our case, we see that a faster processing speed predicts future SA status, but not superior memory performance. This was supported also by additional analysis revealing significant association between CS-speed and SA status, but not between CS-speed and PVLT delayed recall (2018). Based on our data, 36 persons originally fulfilled the delayed memory criteria, however all criteria for the status of SA were fulfilled by 19 persons out of those 36. Therefore, it seems that the SA is not just about youthful memory processes, but retaining normal non-memory performance seems to play an important role either.

Our results did not show an important role of the number of years of education in the prediction of SA, but sex or age, along with processing speed measures (CS-speed), seem to be important in the prediction of SA. As suggested in other SA studies [10, 16, 18, 53], females have higher chance of being SA. Our results support this finding. It may be due to the greater cortical thickness in females compared to males [54]. Also, persons with greater cortical thickness are better able to compensate for cortical thinning [55]. According to our results, younger age was also predictor of SA [56]. It seems that with increasing age the chance of being SA decreases.

## Limitations

The present study has several limitations that need to be acknowledged. We used the PVLT memory test instead of the RAVLT, which was used in the studies performed at the Northwestern Mesulam Center for Cognitive Neurology and Alzheimer’s Disease. Both RAVLT and PVLT are word list tests. The PVLT test is shorter (a 12-word list) than the RAVLT (a 15-word list), and ranks among the categorical word-list tests, such as the CVLT—California Verbal Learning Test [57], which it was modelled after. We found no direct comparisons of RAVLT and PVLT in healthy adults. Nevertheless, CVLT, a test similar to PVLT, was used instead of RAVLT in other SuperAging studies [16, 19] with similar results as in those studies where RAVLT was used [14]. The PVLT is a validated episodic memory test [58] with normative data for older Czech adults [43]. We used a PVLT normative equivalent (with the mean in the age group 60–64 years) that was, coincidentally, the same as for RAVLT in the original studies [9, 10]. The other tests used were the same as in the previous SuperAging studies. Also, our study included quite a small sample because this study was a follow-up to the NANOK, which was not originally designed for this purpose. Despite the fact that there are studies with a larger sample size [34], which is rather an exception in the field of SA studies, our sample size is comparable or even larger compared to most SuperAging studies [14–16, 19]. However, future studies with a larger sample size would be beneficial and could reveal more factors associated with SA status (such as lifestyle characteristics). According to previous study [12], the prevalence of SA is about 10%, therefore future studies should reflect it while planning the sample size.

## Conclusions

The concept of memory SuperAging is a relatively new one. Nevertheless, it has been confirmed by independent research centres. The SA is an ideal status and we tried to understand the role of processing speed in predicting status of SA. The main finding of our study is that SA may be predicted based on their faster processing speed. Thus, it gives support and relevance to the speed of processing concept in aging and show the importance of its monitoring in the older age. Therefore, it seems that monitoring speed of processing may bring us an information not only about decline in cognitive performance, but also it may reflect a future SA status in old-old adults. In future studies, it would be interesting to find modifiable factors for cognitive SuperAging.

## Data Availability

The dataset supporting the conclusions of this article is available in the Open Science Framework page, https://osf.io/wbcer/.
